# Editorial: Precision dentistry and ehealth in oral healthcare

**DOI:** 10.3389/froh.2023.1155166

**Published:** 2023-03-07

**Authors:** Tim Joda, Heiko Spallek

**Affiliations:** ^1^ Clinic of Reconstructive Dentistry, Center of Dental Medicine, University of Zurich, Zurich, Switzerland; ^2^ Department of Reconstructive Dentistry, University Center of Dental Medicine, University of Basel, Basel, Switzerland; ^3^ Faculty of Medicine and Health, The University of Sydney School of Dentistry, Sydney, NSW, Australia

**Keywords:** big data, artificial intelligence, digital health, health information system (HIS), electronic health record (EHR), patient journey, medical-dental integration

**Editorial on the Research Topic** Precision dentistry and ehealth in oral healthcare

The increasing collection of health data coupled with continuous improvement in information processing and analysis have moved us closer to the promises made by precision medicine. However, many barriers still exist when it comes to data fitness that is essential when considering the use of data collected during the regular care process for research. Completeness, accuracy and consistency are three of the main problems researchers are faced with when using data for research (1). We need to enhance data capture in dental Electronic Health Records (EHRs) to adhere to these quality criteria, but also follow the FAIR principles (findable, accessible, interoperable, reusable) (2) and augment the data with information from the medical EHR (lab values, medications, diagnoses). In order to transform unstructured data from clinical notes to structured data that are interoperable and can be processed by analytical algorithms, we will need to harness Artificial Intelligence (AI) methodologies, such as Natural Language Processing (NLP). Finally, clinical information must be synthesized into a format that is useful to clinicians and administrators by employing robust statistical methods to extract, analyze, interpret, and present actionable information. The last step in this process is the development of real-time dashboards that present large amounts of data to all stakeholders, including clinicians, patients, carers, administrators and policymakers while adhering to privacy and confidentiality legislation and patients’ preferences.

When we started the call for this issue, we asked ourselves the uncomfortable question of how far we are from this desired state, or to put it in other words: Water fluoridation has had a significant impact on the prevention of caries. Which dental technology can claim a similar success?

Traditionally, dentistry has lagged behind medicine in the adoption and seamless integration of new technologies, such as precision dentistry and data science that have the potential to improve oral health outcomes. While the articles of this issue showcase technological innovations, we need to acknowledge that, overall, oral health outcomes have not improved in the past two decades which was recently showcased by the US Surgeon General report (3) and in the WHO report (4). Given that tooth decay and tooth loss are not natural consequences of aging but preventable diseases, what has the use of technology in dentistry achieved to improve oral health outcomes? How can technology help reducing the globally approximately 300 years of life lost per 100k people due to issues of oral health? When responding to this provocative question, we should acknowledge the profound mismatch between the human mind's abilities and medicine's complexity. Long ago, we realized that our inborn sensorium was inadequate for scrutinizing the body's inner workings—hence, we developed microscopes, stethoscopes, electrocardiograms, and radiographs. Will our inborn cognition alone solve the mysteries of health and disease? Obermeyer writes in the New England Journal of Medicine: “Medical thinking has become vastly more complex, mirroring changes in our patients, our health care system, and medical science. The complexity of medicine now exceeds the capacity of the human mind” (5).

We believe, and the featured contributions demonstrate, that dentistry is heading in the same direction. While the number of possible diagnosis and treatment options might be lower than in medicine, digital imaging, CAD/CAM restorations and orthodontic sequential aligners would not be possible without informatics. If the scale of decision-making is pushed up to the population level, decisions become even harder to make. Questions such as how certain benefit schemes will change health outcomes can hardly be answered without aggregating and analyzing large amounts of data.

The objective of this Research Topic was to provide an update on the current knowledge with state-of-the-art theory and practical information on precision dentistry and e-health data science in oral healthcare focusing on (i) telemedicine; (ii) digital therapeutics; and (iii) care navigation. This Research Topic comprises 3 Original Research Articles, and 1 Systematic Review. Topics like this one often trigger fears among professional groups that they might get sooner or later replaced by computers following the faith or cartographers or cab drivers.

**Figure 1:**
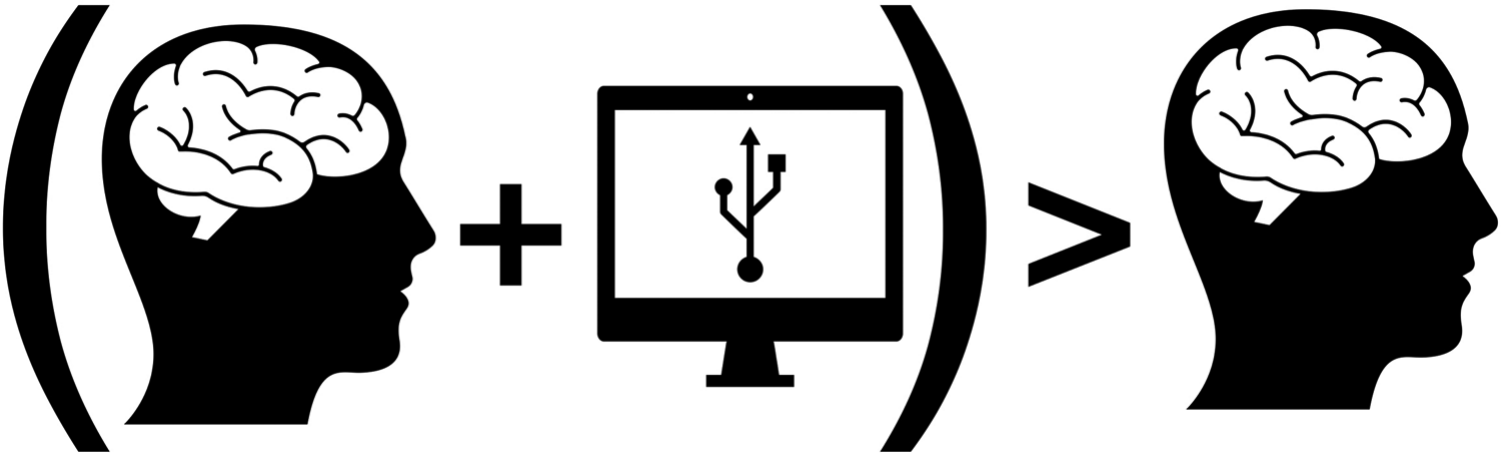
Modified picture originally published by Friedman (6).

We believe it is important to counterbalance this substitution threat, i.e., AI and robotics will replace human clinicians, by always thinking about what dental professionals can do with the help of computers following Chuck P. Friedman's Fundamental Theorem (6) (Figure 1):

As we are aggregating electronic health records data, wide-scale omics information, patient generated health data, and data from environmental sensors, we need to embrace a digital infrastructure that uses AI to make sense of this data to improve the health of our communities. Through data, we will shift healthcare from its focus on diagnosis and treatment to prevention and early intervention—a move from crisis management to health management (7).

EHRs represent a critical foundation for supporting a dental Learning Health System (LHS) (8): Advances in information technology are changing the way health data are collected, especially data obtained at the point of care. The adoption of EHRs in medicine has shown that it is possible to collect medical records from multiple institutions, thus achieving data sets that include millions of individual patients.

The contributing authors’ work makes us optimistic about a future when all health professionals will use Health IT not as an encounter-based reporting tool to support documentation and billing, but rather as a tool to fulfil its original intention: supporting the best possible care for all of our patients—all meaning here:

- Across all socio-economic groups;
- All meaning all encounter types acute, chronic and monitoring wellbeing at home;
- All meaning in hospitals and in ambulatory care settings; and
- All meaning across all geographical areas of all countries.

So, the question is: How do we move such a vision from impossible to imperative to inevitable? The papers in this issue give us a glimpse of what is to come.
